# Urban Heat and Burden of Hyponatremia

**DOI:** 10.1001/jamanetworkopen.2024.50280

**Published:** 2024-12-16

**Authors:** Monika Prpic, Christina Hoffmann, Wolfgang Bauer, Peter Hoffmann, Kai Kappert

**Affiliations:** 1 Institute of Diagnostic Laboratory Medicine, Clinical Chemistry, and Pathobiochemistry, Charité–Universitätsmedizin Berlin, Corporate Member of Freie Universität and Humboldt-Universität zu Berlin, Berlin, Germany; 2 Department of Emergency Medicine, Charité–Universitätsmedizin Berlin, Corporate Member of Freie Universität and Humboldt-Universität zu Berlin, Berlin, Germany

## Abstract

**Importance:**

Hyponatremia (sodium level <135 mEq/L), the most prevalent electrolyte disorder in clinical practice, is associated with considerable clinical and economic burdens. Despite its recognized effect and indication of seasonal patterns, there is a research gap regarding heat-related hyponatremia. Addressing this issue is crucial, especially regarding projected increases in environmental temperature, particularly in urban areas.

**Objective:**

To comprehensively analyze the association of heat in an urban setting with hyponatremia prevalence.

**Design, Setting, and Participants:**

This cross-sectional study using retrospective time series analysis was conducted among all adult patients (age ≥18 years) presenting to the Charité–Universitätsmedizin Berlin between March 1, 2000, and August 31, 2023, with a blood sodium measurement.

**Exposure:**

The daily heat index, which accounts for both outdoor air temperature and relative humidity.

**Main Outcomes and Measures:**

The primary outcome measure was the daily number of hyponatremia cases, further categorized by severity. Differences in prevalence across age groups (adult patients, aged 18-65 years; and older patients, aged >65 years) and sexes were also examined.

**Results:**

A total of 7 135 688 sodium measurements from 2 028 537 hospital visits were analyzed. The mean (SD) age at admission was 57.8 (17.8) years, and 51.7% of patients were male. A clear seasonal pattern of heat-related hyponatremia was evident among older patients, especially for moderate (sodium level, 125-129 mEq/L) and severe hyponatremia (sodium level, <125 mEq/L), where the cumulative risk over a lag period of 5 days reached a maximum relative risk (RR) of 1.26 (95% CI, 1.07-1.48) when the heat index reached 30 °C, compared with the temperature at which hyponatremia occurred least frequently. Older women were disproportionately more likely to experience hyponatremia compared with older men, with a cumulative RR of 1.10 (95% CI, 1.03-1.18) at a heat index of 26 °C. The highest RR was on lag day 0 for all subgroups (older patients: RR, 1.04 [95% CI, 1.00-1.08]; moderate and severe hyponatremia in older patients: RR, 1.05 [1.01-1.10]; and older women: RR, 1.07 [95% CI, 1.01-1.12]) and significantly increased when the heat index exceeded 15 °C (RR, 1.01 [95% CI, 1.00-1.02]).

**Conclusions and Relevance:**

This cross-sectional study of patients with sodium measurements suggests that older people, especially women, were vulnerable to heat-related hyponatremia. Environmental heat was associated with an immediate exacerbation of hyponatremia. This finding highlights the importance of implementing prevention strategies to mitigate heat-related hyponatremia, as an increased burden in the future due to climate change is likely.

## Key Points

**Question:**

Is there an association between short-term exposure to high temperatures and a seasonal-related prevalence of hyponatremia?

**Findings:**

In this cross-sectional study of 2 028 537 patients who were admitted to the hospital and had blood sodium measurements, heat was associated with an increase in hyponatremia cases, especially among individuals older than 65 years. This association was more pronounced in moderate to severe cases of hyponatremia and among older women compared with older men.

**Meaning:**

The findings underscore the importance of implementing prevention strategies, which include education on hydration and sodium monitoring, to reduce the clinical and economic burdens of heat-related hyponatremia.

## Teaser

This cross-sectional study analyzes the association of short-term exposure to high temperatures in an urban setting with the prevalence of hyponatremia.

## Introduction

Extreme weather conditions, including high temperatures, represent a hazard to human health and well-being. Excessive heat often worsens preexisting conditions and can lead to increased morbidity and mortality.^1^ Europe witnessed a heat wave in 2003 that was estimated to be responsible for over 70 000 deaths.^2^ Furthermore, Ballester et al^3^ reported that over 61 000 Europeans lost their lives in the summer of 2022 due to extreme heat. Heat stress represents a major concern, primarily for urban areas due to steadily increasing urbanization and the urban heat island effect, which contributes to higher daytime temperatures and reduced nighttime cooling.

As extreme heat linked with climate change is likely to become more frequent and intense in the future,^4^ it is necessary to identify heat-related mechanisms, including changes in hydration, involved in developing electrolyte imbalance (hypernatremia or hyponatremia). Noakes and colleagues^5^ reported that hyponatremia—caused by an excess of total body water compared with total body sodium content—occurs in endurance athletes due to overconsumption of hypotonic drinks, and increased temperatures could lead to the same scenario. Nonetheless, environmental health studies have traditionally focused on overall mortality or prevalent health outcomes, with few having addressed heat stress in the context of electrolyte homeostasis and laboratory medicine. A systematic review confirmed the association between higher ambient temperatures and increased incidence of hyponatremia.^6^ However, significant limitations remain, including the retrospective nature of most previous studies, high variability in sample sizes, and lack of consideration of potentially predisposing comorbidities and relevant pathophysiological factors. Furthermore, there are limited detailed quantitative data. Although not all these limitations were addressed, our analysis delved deeper into heat-related hyponatremia, incorporating factors such as comorbidities, age, and sex.

Hyponatremia is the most common electrolyte disorder in clinical practice,^7,8,9^ with symptoms ranging from mild and unspecific, such as headache, nausea, fatigue, and confusion, to life-threatening cerebral edema. The cause of hyponatremia varies and is often multifactorial.^6,10^ Polydipsia, excessive water consumption from, for example, sweating, diabetes, or kidney diseases, can cause true (hypotonic) hyponatremia.^6^ Also, certain medications, including diuretics, angiotensin-converting enzyme inhibitors, and some antidepressants, can be associated with hyponatremia.^10,11^ Furthermore, endocrine diseases, particularly the syndrome of inappropriate antidiuresis (SIAD), are frequent causes, with SIAD stemming from malignant neoplasms or pulmonary or central nervous system pathologic conditions.^12^ Thus, hyponatremia results from sodium loss, impaired water excretion, or excessive water intake. However, in our study, heat was the primary variable that seasonally changed over the years and that was addressed in depth.

Hyponatremia has been widely recognized as independently associated with morbidity and mortality, particularly among older individuals and vulnerable populations.^7,8,9,13,14,15^ Previous studies have reported seasonal variations of hyponatremia, with a higher prevalence during the summer,^16,17,18,19^ especially among older individuals.^16,17,18,20,21,22^ This greater susceptibility is explained mainly by impaired water-excretory capacity and altered sensation of thirst or actively overcompensated thirst. Furthermore, frequent exposure to medications and diseases can contribute to hyponatremia. Also, while previous publications identified women as a risk group, the reasons for the sex disparity remain unclear.^8,16,18,20,21^ Last, the clinical burden caused by this seasonal phenomenon is highlighted. Hyponatremia is associated with prolonged length of hospital stay and higher risk of readmission.^23^ Furthermore, in many European cities, air conditioning in hospitals is uncommon. This includes in Charité Hospital, where regular patient rooms and the emergency department typically lack temperature control.^24^

Thus, a better understanding of the underlying causes could help minimize or even prevent hyponatremia, reduce associated complications and hospitalization costs, and improve resource allocation. This study aimed to systematically investigate the association between increasing temperatures and the prevalence of hyponatremia in Berlin, Germany.

## Methods

### Study Design

In this cross-sectional study, we conducted a retrospective time series analysis including all adult patients (age ≥18 years) presenting to the Charité–Universitätsmedizin Berlin, one of the largest university hospitals in Europe, between March 1, 2000, and August 31, 2023, with a blood sodium measurement. The study was performed according to the Declaration of Helsinki^25^ and was approved by the ethics committee and the data protection officer of the Charité–Universitätsmedizin Berlin. Patients’ consent was not required by the ethics committee, because section 25 of the Berlin Hospital Law (Berliner Landeskrankenhausgesetz) allows the usage of hospital patient data after anonymization for research purposes. This study followed the Strengthening the Reporting of Observational Studies in Epidemiology (STROBE) reporting guideline.

### Data Collection

Patients’ electronic health records, containing all data relevant to the study, were retrieved from the Health Data Platform system, which is responsible for hosting patient data from the Charité Hospital. We gathered information regarding patients’ age, sex, admission date and department, length of stay, and main diagnosis. In addition to demographic and clinical data, we also included an extensive overview of the laboratory test parameters.

### Exposure Variable

The daily time series of meteorologic data for the Berlin-Tempelhof weather station in the urban center of the city were obtained from the Deutscher Wetterdienst.^26^ In addition, we considered various indices for measuring thermal comfort. Finally, we identified the heat index, which combines outdoor air temperature and relative humidity, as the best factor for assessing the risk of hyponatremia due to nonoptimal temperature.^27^

### Outcome Variable

The primary outcome of interest for this analysis was the daily number of patients who experienced hyponatremia, defined as a serum sodium level less than 135 mEq/L (to convert to millimoles per liter, multiply by 1.0). Hyponatremia was further categorized by its severity using the following cutoffs: sodium level 130 to 135 mEq/L for mild hyponatremia, sodium level 125 to 129 mEq/L for moderate hyponatremia, and sodium level less than 125 mEq/L for severe hyponatremia.^7^ Furthermore, cases of pseudohyponatremia due to hyperglycemia were corrected.^28^

The sodium level of some patients was measured multiple times throughout the same day. For those hospitalized over several days, hyponatremia status was reevaluated daily when a sodium measurement was available. To avoid inflated rates from repeated daily measurements, we considered the lowest recorded value for each day as relevant for risk assessment purposes.

### Statistical Analysis

Continuous variables are presented as the mean (SD) or median (IQR), as appropriate, and categorical data are presented as counts and percentages. To estimate the temperature-hyponatremia association, we used a quasi-Poisson time series regression. This model was further coupled with a distributed lag nonlinear model to capture both the nonlinear exposure-response association and possible delayed association through a cross-basis function. Specifically, we selected a cross-basis composed of a quadratic B-spline for the exposure response with 2 internal knots placed at the 50th and 90th percentiles of the daily mean heat index distribution to address the association of heat with hyponatremia. The lag response was modeled as a natural cubic spline with 2 internal knots placed at equally spaced values in the log scale and was extended to 5. We applied a sandwich estimator to correct the variance-covariance matrix of heat index to ensure the robustness of SEs to autocorrelation and heteroskedasticity.^29,30^

We controlled for seasonality and long-term trends with natural cubic spline of time with 7 *df* per year. The models were further adjusted for the day of the week and public holidays in Berlin. To account for demographic characteristic changes and health care dynamics over the 23-year study period, we incorporated the monthly number of cases with sodium measurement for each respective year as a proxy for the population in our models. In addition, we controlled for comorbidities linked with hyponatremia by including person-day cases with the following main diagnoses: infectious diseases, circulatory diseases, liver diseases, malignant neoplasms, kidney failure, and respiratory diseases.

Optimal heat index, used as the reference to calculate relative risk (RR) ratios, was derived from the cumulative exposure-response curve over the 5-day lag period as the minimum between the median and maximum heat index. Sensitivity analyses, testing different model configurations, are presented in eTable 2 in Supplement 1.

The best-fitting models were selected based on minimizing the quasi-Akaike information criterion. A 2-sided *P* < .05 was considered statistically significant. Analysis was performed using R software, version 4.2.2, and the dlnm package (R Project for Statistical Computing).^31^

## Results

### Patients’ Characteristics

Our study investigated a total of 2 028 537 patient admissions. The mean (SD) age at admission was 57.8 (17.8) years; 51.7% of the patients were male and 48.3% of the patients were female. The baseline characteristics of the patients are summarized in Table 1. The overall prevalence of hyponatremia was 15.4%. There were 618 850 admissions at the emergency department, where the prevalence of hyponatremia was 20.4%—significantly higher than the 13.1% observed among hospital admissions to other departments (odds ratio [OR], 1.70 [95% CI, 1.69-1.71]; *P* < .001). A higher number of cases with hyponatremia was also observed among inpatients compared with outpatients (OR, 2.66 [95% CI, 2.63-2.69]; *P* < .001); inpatient stay was characterized by a duration of hospital stay longer than 24 hours. The most common main comorbidities in the hyponatremia group, which also carried a higher OR, are outlined in Table 1.

**Table 1.** Baseline Characteristics

Characteristic	No. (%)		Odds ratio (95% CI)^a^
	Without hyponatremia (n = 1 717 018)	With hyponatremia (n = 311 519)	
Age at admission, mean (SD), y	56.9 (17.9)	62.8 (16.4)	NA
Age group, y			
≤65	1 069 236 (62.3)	152 962 (49.1)	1 [Reference]
>65	647 782 (37.7)	158 557 (50.9)	1.71 (1.70-1.72)
Sex			
Female	829 832 (48.3)	149 756 (48.1)	1 [Reference]
Male	887 186 (51.7)	161 763 (51.9)	1.01 (1.00-1.02)
Department of admission			
Emergency department	492 751 (28.7)	126 099 (40.5)	1.70 (1.69-1.71)
Other department	1 190 865 (69.3)	179 367 (57.6)	1 [Reference]
Missing	33 402 (2.0)	6053 (1.9)	NA
Type of medical care			
Inpatient	1 228 017 (71.5)	268 068 (86.1)	2.66 (2.63-2.69)
Outpatient	455 593 (26.5)	37 398 (12.0)	1 [Reference]
Missing	33 408 (1.9)	6053 (1.9)	NA
Main diagnosis^b^			
Kidney failure	19 245 (1.1)	8768 (2.8)	2.20 (2.14-2.26)
Liver diseases	18 053 (1.1)	7571 (2.4)	2.01 (1.96-2.07)
Infectious and parasitic diseases	39 894 (2.3)	16 367 (5.3)	2.00 (1.97-2.04)
Diseases of the respiratory system	48 137 (2.8)	16 034 (5.1)	1.61 (1.58-1.64)
Malignant neoplasms	26 1831 (15.2)	74 142 (23.8)	1.47 (1.45-1.48)
Diseases of the circulatory system	201 580 (11.7)	44 428 (14.3)	1.05 (1.04-1.06)
Other diseases of the digestive system	109 370 (6.4)	22 078 (7.1)	0.95 (0.93-0.97)
Endocrine, nutritional, and metabolic diseases	50 376 (2.9)	9290 (3.0)	0.87 (0.85-0.89)
Diseases of the blood and blood-forming organs and certain disorders involving the immune mechanism	14 080 (0.8)	2367 (0.8)	0.79 (0.76-0.83)
Diseases of the genitourinary system	54 650 (3.2)	7856 (2.5)	0.67 (0.66-0.69)
Diseases of the nervous system	66 038 (3.8)	8412 (2.7)	0.59 (0.58-0.60)
Other neoplasms	33 810 (2.0)	4316 (1.4)	0.60 (0.58-0.62)
Mental, behavioral, and neurodevelopmental disorders	56 454 (3.3)	4242 (1.4)	0.35 (0.34-0.35)
No main diagnosis coded	416 276 (24.2)	36 926 (11.9)	NA
Other	327 224 (19.1)	48 722 (15.6)	NA

Abbreviation: NA, not applicable.

^a^ Odds ratios with 95% CIs were calculated using the Fisher exact test.

^b^ The main diagnosis was coded according to the *International Statistical Classification of Diseases and Related Health Problems, 10th revision, German Modification*.

### Monthly Variations in the Prevalence of Hyponatremia

**Figure 1.**
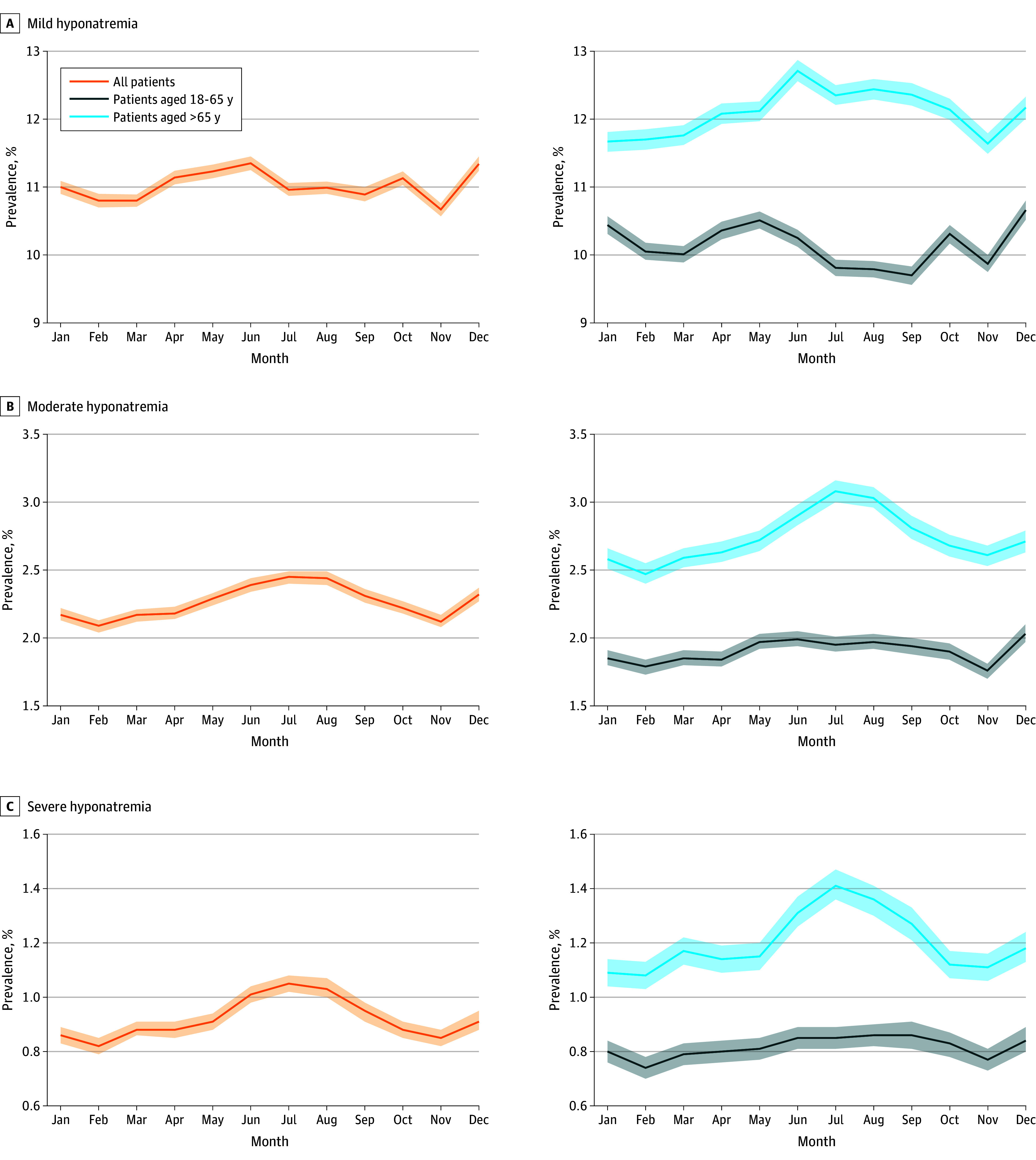
Monthly Variations in the Prevalence of Hyponatremia Age-related monthly variations in prevalence of mild (sodium level, 130-135 mEq/L), moderate (sodium level, 125-129 mEq/L), and severe (sodium level, <125 mEq/L) (to convert to millimoles per liter, multiply by 1.0) hyponatremia as outlined in the Materials and Methods section. Solid lines depict monthly rates and the shaded areas represent 95% CIs estimated using the Agresti-Coull interval method.

To account for variation in prevalence rates of hyponatremia across age groups, we categorized patients into 2 groups: adults (age, 18-65 years) and older adults (age, >65 years). Figure 1 shows mild hyponatremia as more resistant to seasonal changes, while moderate and severe conditions tend to increase during the summer months (June-August), particularly among older patients. Older patients exhibit a higher prevalence of hyponatremia, peaking in July with increases of 5.6% for mild cases, 24.7% for moderate cases, and 30.6% for severe cases, compared with the lowest rates, which were typically reported in February (eTable 1 in Supplement 1). Furthermore, monthly variations among older adults were significantly correlated with the heat index, unlike in the adult group (eFigure 1 in Supplement 1).

Although we detected neither a clear trend in monthly variations of hyponatremia for either sex in the adult patients’ group, nor a consistent difference between them, analysis of the subgroup of older patients yielded more prominent results. Older women were more susceptible to all grades of hyponatremia, especially during the summer. In July, prevalence of hyponatremia increased by 17.0% for mild cases, 54.1% for moderate cases, and 66.4% for severe cases compared with men of the same age (eFigure 2 and eTable 1 in Supplement 1).

### Laboratory Data

During the study period, we retrieved a total of 7 135 688 sodium measurements from 2 028 537 patient admissions. An overview of the laboratory test parameters is presented in Table 2. Measured blood analytes were grouped into clinical chemistry analysis, hematologic analysis, hemostaseology, and others. We observed no clinically significant differences in potassium, calcium, and magnesium levels between patients with and without hyponatremia. Hyponatremia was not associated with major alterations in hematologic parameters, arguing against severe dehydration or hyperhydration. In addition, our analysis revealed no substantial differences in kidney function parameters between patients with hyponatremia and those without. Among patients who developed hyponatremia, the median C-reactive protein value was notably higher (4.3 mg/dL [95% CI, 1.2-10.3 mg/dL]) compared with those without hyponatremia (1.3 mg/dL [95% CI, 0.3-5.3 mg/dL]) (to convert to milligrams per liter, multiply by 10.0). Patients with hyponatremia had lower median chloride levels than patients without hyponatremia (101 mEq/L [95% CI, 96-104 mEq/L] vs 108 mEq/L [95% CI, 105-111 mEq/L]) (to convert to millimoles per liter, multiply by 1.0).

**Table 2.** Summary of the Laboratory Data

Analysis	Laboratory values	
	Without hyponatremia (n = 6 276 669)	With hyponatremia (n = 859 019)
**Clinical chemistry analysis**		
Potassium, mEq/L		
Median (IQR)	4.1 (3.8-4.4)	4.2 (3.8-4.6)
No. (%)	6 248 769 (99.6)	842 293 (98.1)
Calcium, mg/dL		
Median (IQR)	7.7 (4.8-9.2)	7.5 (4.6-8.9)
No. (%)	1 492 717 (23.8)	203 390 (23.7)
Chloride, mEq/L		
Median (IQR)	108 (105-111)	101 (96-104)
No. (%)	3 307 215 (52.7)	364 902 (42.5)
Magnesium, mg/dL		
Median (IQR)	2.0 (1.8-2.1)	1.9 (1.6-2.1)
No. (%)	289 455 (4.6)	42 010 (4.9)
AST, U/L		
Median (IQR)	26.0 (20.0-36.0)	30.0 (21.0-54.0)
No. (%)	1 578 576 (25.1)	221 861 (25.8)
ALT, U/L		
Median (IQR)	23.0 (16.0-38.0)	25.0 (15.0-48.0)
No. (%)	1 622 694 (25.9)	229 280 (26.7)
GGT, U/L		
Median (IQR)	37.0 (20.0-91.0)	77.0 (33.0-206.0)
No. (%)	578 705 (9.2)	98 110 (11.4)
CRP, mg/dL		
Median (IQR)	1.3 (0.3-5.3)	4.3 (1.2-10.3)
No. (%)	1 817 313 (29.0)	317 818 (37.0)
CK, U/L		
Median (IQR)	88 (54-150)	81 (41-167)
No. (%)	509 851 (8.1)	63 117 (7.3)
CK-MB, U/L		
Median (IQR)	17 (13-24)	19 (13-31)
No. (%)	144 254 (2.3)	21 653 (2.5)
Glucose, mg/dL		
Median (IQR)	126 (104-157)	121 (102-147)
No. (%)	3 610 838 (57.5)	404 387 (47.1)
Creatinine, mg/dL		
Median (IQR)	0.9 (0.7-1.2)	0.9 (0.7-1.4)
No. (%)	2 928 993 (46.7)	465 704 (54.2)
eGFR, mL/min/1.73 m^2^		
Median (IQR)	71.0 (50.0-86.0)	65.0 (43.0-85.0)
No. (%)	1 149 514 (18.3)	152 438 (17.7)
**Hematologic analysis**		
Leukocytes, cells/µL		
Median (IQR)	7180 (5350-9530)	7790 (5190-11 000)
No. (%)	2 597 600 (41.4)	389 118 (45.3)
Thrombocytes, cells × 10^3^/µL		
Median (IQR)	231 (170-297)	227 (138-321)
No. (%)	2 609 649 (41.6)	389 875 (45.4)
Erythrocytes, cells × 10^6^/µL		
Median (IQR)	4.1 (3.4-4.6)	3.6 (3.1-4.1)
No. (%)	2 599 126 (41.4)	389 801 (45.4)
Hematocrit, %		
Median (IQR)	0.360 (0.303-0.410)	0.312 (0.270-0.360)
No. (%)	2 615 727 (41.7)	391 472 (45.6)
Hemoglobin, g/dL		
Median (IQR)	12.0 (10.0-13.8)	10.5 (9.10-12.2)
No. (%)	2 618 491 (41.7)	394 208 (45.9)
MCH, pg/cell		
Median (IQR)	29.9 (28.6-31.2)	29.8 (28.4-31.2)
No. (%)	2 613 619 (41.6)	391 008 (45.5)
MCHC, g/dL		
Median (IQR)	33.4 (32.5-34.3)	33.8 (32.8-34.8)
No. (%)	2 613 583 (41.6)	390 924 (45.5)
MCV, µm^3^		
Median (IQR)	89.0 (86.0-93.0)	88.0 (84.0-92.0)
No. (%)	2 613 360 (41.6)	391 030 (45.5)
MPV, µm^3^		
Median (IQR)	10.4 (10.0-11.0)	10.0 (9.60-11.0)
No. (%)	2 531 121 (40.3)	368 531 (42.9)
**Hemostaseology**		
INR		
Median (IQR)	1.06 (0.99-1.17)	1.13 (1.04-1.29)
No. (%)	1 450 135 (23.1)	229 322 (26.7)
**Further analysis**		
Blood urea nitrogen, mg/dL		
Median (IQR)	15.4 (11.2-23.8)	17.3 (10.7-30.4)
No. (%)	1 787 213 (28.5)	290 157 (33.8)
Osmolality, mOsm/kg		
Median (IQR)	289 (285-294)	276 (270-281)
No. (%)	263 656 (4.2)	42 635 (5.0)

Abbreviations: ALT, alanine aminotransferase; AST, aspartate aminotansferase; CK, creatine kinase; CK-MB, creatine kinase–MB fraction; CRP, C-reactive protein; eGFR, estimated glomerular filtration rate; GGT, γ-glutamyl transferase; INR, international normalized ratio; MCH, mean corpuscular hemoglobin; MCHC, mean corpuscular hemoglobin concentration; MCV, mean corpuscular volume; MPV, mean platelet volume.

SI conversion factors: To convert potassium to millimoles per liter, multiply by 1.0; calcium to millimoles per liter, multiply by 0.25; chloride to millimoles per liter, multiply by 1.0; magnesium to millimoles per liter, multiply by 0.4114; AST, ALT, GGT, and CK to microkatals per liter, multiply by 0.0167; CRP to milligrams per liter, multiply by 10.0; glucose to millimoles per liter, multiply by 0.0555; creatinine to micromoles per liter, multiply by 88.4; leukocytes to 10^9^ cells per liter, multiply by 0.001; thrombocytes to 10^9^ cells per liter, multiply by 1.0; erythrocytes to 10^12^ cells per liter, multiply by 1.0; hematocrit to proportion of 1.0, multiply by 0.01; hemoglobin to grams per liter, multiply by 10.0; MCHC to grams per liter, multiply by 10; MCV to femtoliters, multiply by 1.0; MPV to femtoliters, multiply by 1.0; blood urea nitrogen to millimoles per liter, multiply by 0.357; and osmolality to millimoles per kilogram, multiply by 1.0.

### Variation in Risk Between Susceptible Subgroups

The cumulative exposure-response curve showing the association between daily mean heat index and hyponatremia prevalence among older individuals is illustrated in eFigure 3A in Supplement 1. The curve increases steadily until reaching a heat index of 23 °C—which corresponds to the 95.5th percentile of the daily mean heat index—compared with the baseline of 13 °C, where hyponatremia occurrence was lowest. A similar curve for adults justified excluding them from further risk assessment (eFigure 3B in Supplement 1).

**Figure 2.**
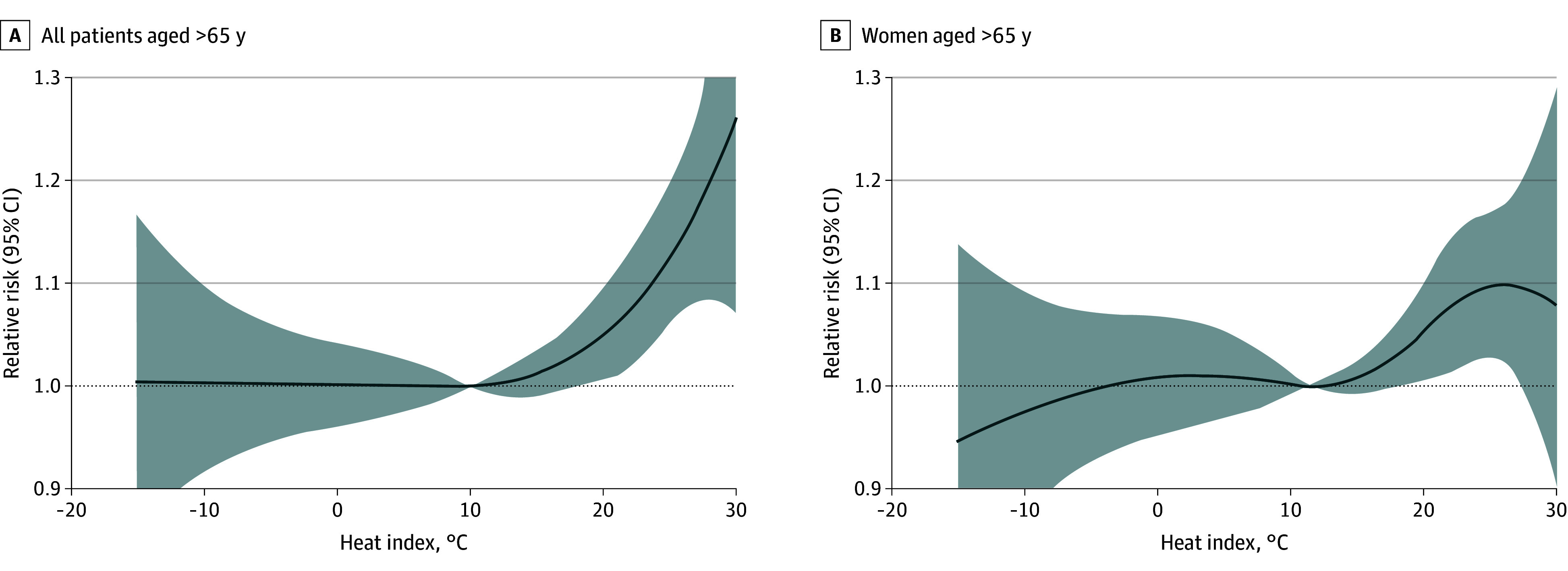
Relative Risk of Hyponatremia in Association With the Heat Index Cumulative relative risk ratios of moderate and severe hyponatremia associated with the heat index among all older patients (age, >65 y) and older women regardless of severity of hyponatremia.

A model for moderate and severe hyponatremia, particularly in older patients, yielded a monotonically increasing exposure-response curve, with RRs peaking at 1.26 (95% CI, 1.07-1.48) when the heat index reached 30 °C (Figure 2A). Similarly, quantifying the risk in older women—identified as the most susceptible subgroup—resulted in a cumulative RR of 1.10 (95% CI, 1.03-1.18) at a heat index of 26 °C (Figure 2B).

**Figure 3.**
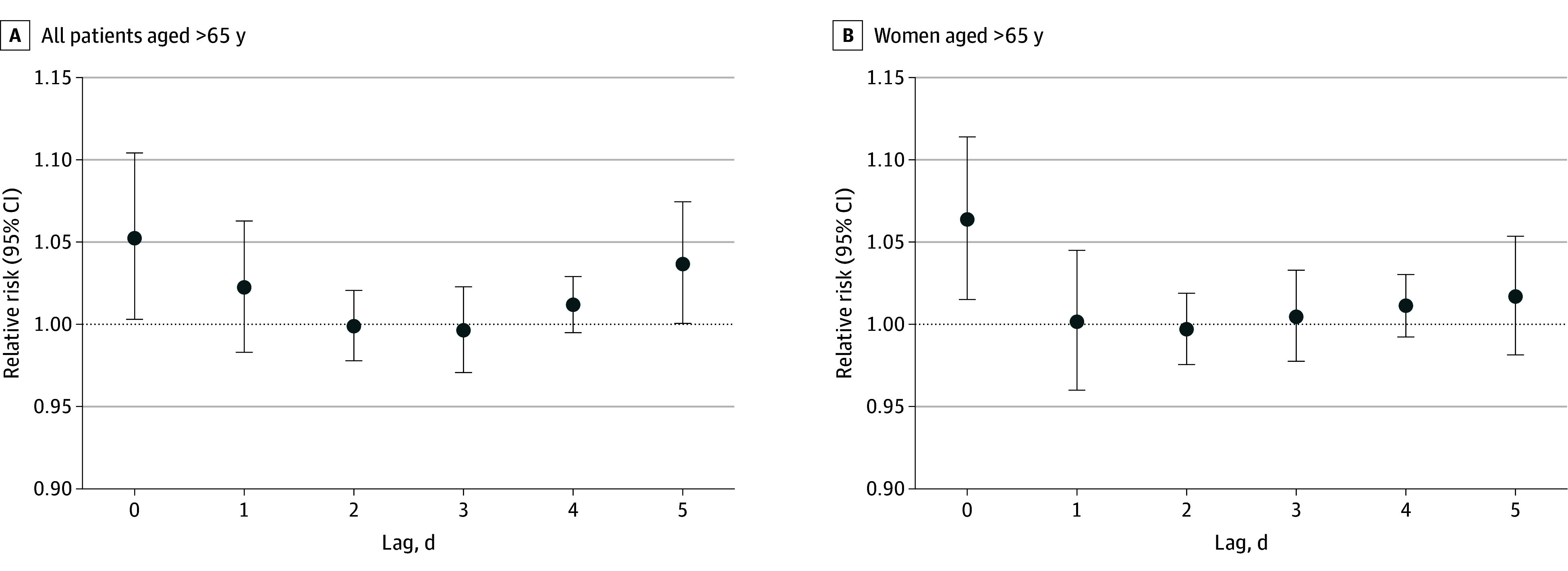
Distribution of the Risk of Hyponatremia Across Lag Days 0 to 5 Distribution of the risk of moderate and severe hyponatremia across lag days 0 to 5 among all older patients (age, >65 y) and older women regardless of severity of hyponatremia.

The RRs peaked on lag day 0 for a heat index of 25 °C (Figure 3; eFigure 4 in Supplement 1), suggesting immediate exacerbation of hyponatremia by a nonoptimal heat index. Relative risks were 1.04 (95% CI, 1.00-1.08) for older patients (eFigure 4A in Supplement 1), 1.05 (95% CI, 1.01-1.10) for older patients with moderate and severe hyponatremia (Figure 3A), and 1.07 (95% CI, 1.01-1.12) for older women (Figure 3B). Risks remained slightly elevated for subsequent days but were not statistically significant. A heat index of 15 °C is considered a threshold for provoking hyponatremia in older individuals, as it was the lowest statistically significant point (RR, 1.01 [95% CI, 1.00-1.02]).

### Sensitivity Analyses

We performed sensitivity analyses to inspect the robustness of the main models. None of the adjustments to the model significantly altered the results (eTable 2 in Supplement 1).

## Discussion

We investigated the association between high temperatures and hyponatremia in Berlin, Germany, with a focus on age-related and sex-related differences. To our knowledge, this is the largest and most comprehensive study of heat-related hyponatremia and the first in Germany to delve into this topic. It examines the issue on a scale broader than that of previous studies, which concentrated largely on specific clinical settings or smaller patient cohorts. Our findings are consistent with previous studies and suggest that nonoptimal temperature conditions are associated with a substantial fraction of hyponatremia cases, mainly among older individuals, where the risk can increase by up to 26% if the heat index exceeds the optimal value.

Considering the aging population, addressing the topic of hyponatremia is of increasing importance. Aging decreases the body’s tolerance to heat stress and may compromise body fluid regulation via multiple mechanisms. The thermoregulatory response to increasing ambient temperatures through sweating declines with age, as does the ability to excrete free water.^32^ Furthermore, older individuals often experience altered thirst sensation.^33^ However, among older individuals under nursing care, this altered thirst sensation can also potentially provoke enhanced or overcompensating hypotonic fluid intake.^34^ Furthermore, medications and diseases associated with hyponatremia should not be overlooked.^11^ Older people are predisposed to hyponatremia due to comorbidities such as kidney diseases, infections, and malignant neoplasms,^35^ which aligns with our findings.

We observed a significant increase in the risk of hyponatremia once the heat index exceeded 15 °C, peaking at 25 °C. This increase might be due to the prevalence of mild hyponatremia, which dominates overall rates. As temperatures progress toward extremes, those at risk of hyponatremia tend to experience more profound decreases in sodium levels, turning mild hyponatremia to moderate or severe hyponatremia. Earlier studies have also indicated that milder but nonoptimal temperatures pose a higher mortality risk than extreme temperatures.^36^ Our findings also align with a Swedish study that identified 15 °C as a critical temperature threshold for hyponatremia.^21^

Furthermore, lack of an association between heat and mild hyponatremia might be due to cases of mild hyponatremia being secondary findings in patients visiting the hospital for other reasons. During summer, when planned interventions and routine visits might decrease, the detection rate of mild hyponatremia might also decline. However, hospital wards remain operational throughout the summer, ensuring that symptomatic hyponatremia cases are still identified.

In addition, laboratory test results revealed no major differences in hemostaseologic, hematologic, and most electrolyte parameters between patients with and without hyponatremia. Lower chloride levels, however, were more often present in patients with hyponatremia, underlining synchronized regulation. No major differences in kidney function parameters were found between the 2 groups. Although impaired kidney function per se predisposes patients to hyponatremia, our data suggest that patients at risk might be monitored and treated under a closer regimen.

### Limitations

This study has some limitations. First, we could not account for the patients’ living conditions over the study period. Factors such as building characteristics, urban heat island effects, air conditioning, and access to green spaces could influence the variability in experienced heat stress. This is particularly important because heat-related emergencies are more widespread in socially vulnerable and disadvantaged communities.^37^ Demographic changes in Berlin indicate that the general quality of living as well as the health status of Berlin residents has improved within the investigation period, possibly affecting our results. Despite this, the association of heat with hyponatremia remained relatively stable over the 2 decades. To fully understand the role of potential confounders such as comorbidities or medications, a detailed analysis of hyponatremia’s cause, including blood osmolality, is needed. However, with osmolality data available for only 5.2% of the cohort, as well as the lack of systematically recorded medication in the electronic health record, our understanding of these confounding factors is limited.

Also, our dataset includes only the comorbidities for which patients received treatment in the hospital. For clarity, our analysis focused on the primary diagnoses recorded at discharge, which can be influenced by health care coding practices.

Potential sources of bias in our person-day approach includes repeated measurements from the same patient and the resulting intrapatient variability. However, relying solely on the first sodium measurement could result in information loss, as hospital environments are not typically fully protective against the development of hyponatremia. Patients with multiple measurements often have longer stays and may be admitted for reasons other than hyponatremia. Even if patients were not initially admitted with hyponatremia, they could still develop it later during their hospitalization, particularly if weather conditions change and they are subjected to pathophysiological changes that predispose them to hyponatremia. Hence, we intentionally included cases where multiple sodium measurements were available because they provide valuable insights into the progression and variability of hyponatremia during hospitalization and help address the phenomenon of hospital-acquired hyponatremia.

Furthermore, blood sodium measurements are not always ordered by the clinician in charge, especially for patients with mild or asymptomatic cases, possibly underestimating the prevalence of hyponatremia. Last, the potential underreporting of relevant cases could have occurred due to several changes in the laboratory information systems at the Charité Hospital over 24 years. Data migrations from older systems to the current system might have been disrupted at some stage, resulting in the loss of some records. However, sensitivity analyses showed only slight changes when excluding specific periods that potentially contained incomplete laboratory data.

## Conclusions

In this cross-sectional study of patients with sodium measurements, older individuals, particularly women, were found to be more susceptible to heat-related hyponatremia, especially its moderate and severe forms. Moderate heat (heat index ≥15 °C) exacerbates sodium disturbances, with nonoptimal heat exposure acting as an immediate trigger. Hyponatremia, although preventable, imposes clinical and economic burdens on health care, a challenge likely to intensify with increasing temperatures. Understanding mechanisms and raising awareness among vulnerable populations can aid in prevention and reduce hospital burdens. This study underscores the need for targeted prevention strategies, including education on drinking behaviors and regular sodium monitoring, particularly in facilities dealing with at-risk populations such as patients with dementia, including nursing homes and geriatric wards. Enhanced regulatory measures and awareness in health care, such as promoting hydration balance, monitoring early symptoms, and ensuring proper temperature control, are crucial to mitigate heat-related hyponatremia.

## Notes

Supplement 1. **eFigure 1.** Association Between Mean Monthly Heat Index and Prevalence Rates, Stratified by Age Group **eFigure 2.** Sex-Related Monthly Variations in Prevalence of Mild (Na^+^ 130-135 mmol/L), Moderate (Na^+^ 125-129 mmol/L), and Severe (Na^+^ <125 mmol/L) Hyponatremia as outlined in the Materials and Methods Section **eFigure 3.** Cumulative Risk Ratios (RR) of Hyponatremia due to Increasing Heat Index for Elderly (A), and Adults (B) **eFigure 4.** Distribution of the Risk Across Lag Days 0 to 5 in Elderly (A), and Adults (B) **eTable 1.** Monthly Summary of Meteorological Variables and Prevalence Rates **eTable 2.** Sensitivity Analyses of the Relative Risk and 95% Confidence Interval in Elderly, Compared to the Baseline HI at Which Hyponatremia Occurred Least Frequently and Cumulated Over a 5-Day Lag Period (With the Exception of Cases M5 and M6, Where We Adjusted the Number of Lag Days)

Supplement 2.
Data Sharing Statement
