# Supplementary material for: Urban Heat and Burden of Hyponatremia
Source: JAMA Netw Open. 2024 Dec 16;7(12):e2450280. doi: 10.1001/jamanetworkopen.2024.50280 (PMC11650395; doi:10.1001/jamanetworkopen.2024.50280)
Supplement: Supplement 1. — eFigure 1. Association Between Mean Monthly Heat Index and Prevalence Rates, Stratified by Age Group eFigure 2. Sex-Related Monthly Variations in Prevalence of Mild (Na+ 130-135 mmol/L), Moderate (Na+ 125-129 mmol/L), and Severe (Na+ <125 mmol/L) Hyponatremia as outlined in the Materials and Methods Section eFigure 3. Cumulative Risk Ratios (RR) of Hyponatremia due to Increasing Heat Index for Elderly (A), and Adults (B) eFigure 4. Distribution of the Risk Across Lag Days 0 to 5 in Elderly (A), and Adults (B) eTable 1. Monthly Summary of Meteorological Variables and Prevalence Rates eTable 2. Sensitivity Analyses of the Relative Risk and 95% Confidence Interval in Elderly, Compared to the Baseline HI at Which Hyponatremia Occurred Least Frequently and Cumulated Over a 5-Day Lag Period (With the Exception of Cases M5 and M6, Where We Adjusted the Number of Lag Days) [file jamanetwopen-e2450280-s001.pdf]

Supplementary Online Content

Prpic M, Hoffmann C, Bauer W, Hoffmann P, Kappert K. Urban heat and burden of hyponatremia. *JAMA Netw Open.* 2024;7(12):e2450280. doi:10.1001/jamanetworkopen.2024.50280

eFigure 1. Association Between Mean Monthly Heat Index and Prevalence Rates, Stratified by Age Group

eFigure 2. Sex-Related Monthly Variations in Prevalence of Mild (Na^+ 130-135 mmol/L), Moderate (Na^+ 125-129 mmol/L), and Severe (Na^+ < 125 mmol/L) Hyponatremia as outlined in the Materials and Methods Section

eFigure 3. Cumulative Risk Ratios (RR) of Hyponatremia due to Increasing Heat Index for Elderly (A), and Adults (B)

eFigure 4. Distribution of the Risk Across Lag Days 0 to 5 in Elderly (A), and Adults (B)

eTable 1. Monthly Summary of Meteorological Variables and Prevalence Rates

eTable 2. Sensitivity Analyses of the Relative Risk and 95% Confidence Interval in Elderly, Compared to the Baseline HI at Which Hyponatremia Occurred Least Frequently and Cumulated Over a 5-Day Lag Period (With the Exception of Cases M5 and M6, Where We Adjusted the Number of Lag Days)

This supplementary material has been provided by the authors to give readers additional information about their work.

© 2024 Prpic M et al. *JAMA Network Open.*

eFigure 1. Association Between Mean Monthly Heat Index and Prevalence Rates, Stratified by Age Group

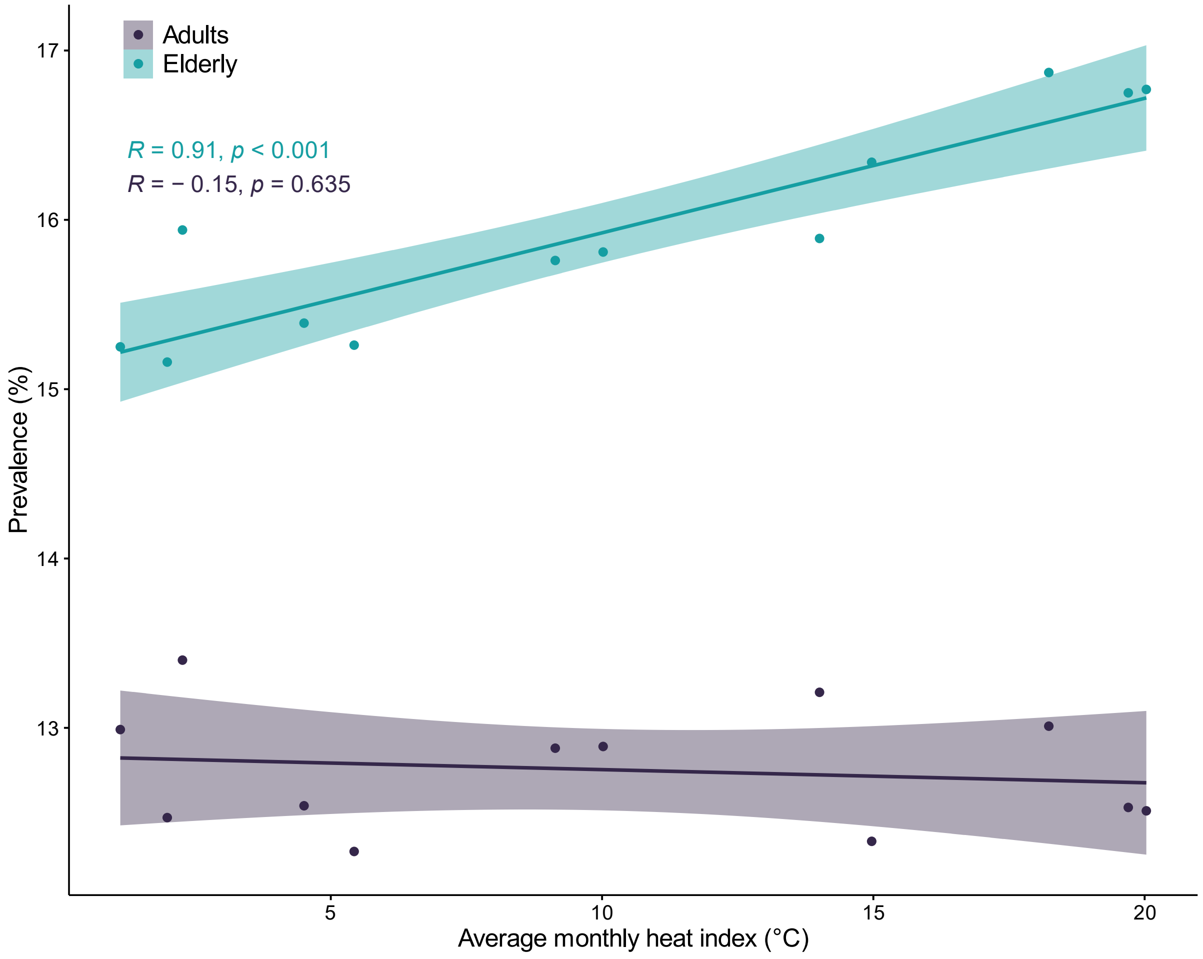

© 2024 Prpic M et al. *JAMA Network Open.*

eFigure 2. Sex-Related Monthly Variations in Prevalence of Mild (Na+ 130-135 mmol/L), Moderate (Na+ 125-129 mmol/L), and Severe (Na+ < 125 mmol/L) Hyponatremia as outlined in the Materials and Methods Section. Solid lines depict monthly rates, and shaded ribbons represent 95% confidence intervals estimated using the Agresti-Coull interval method.

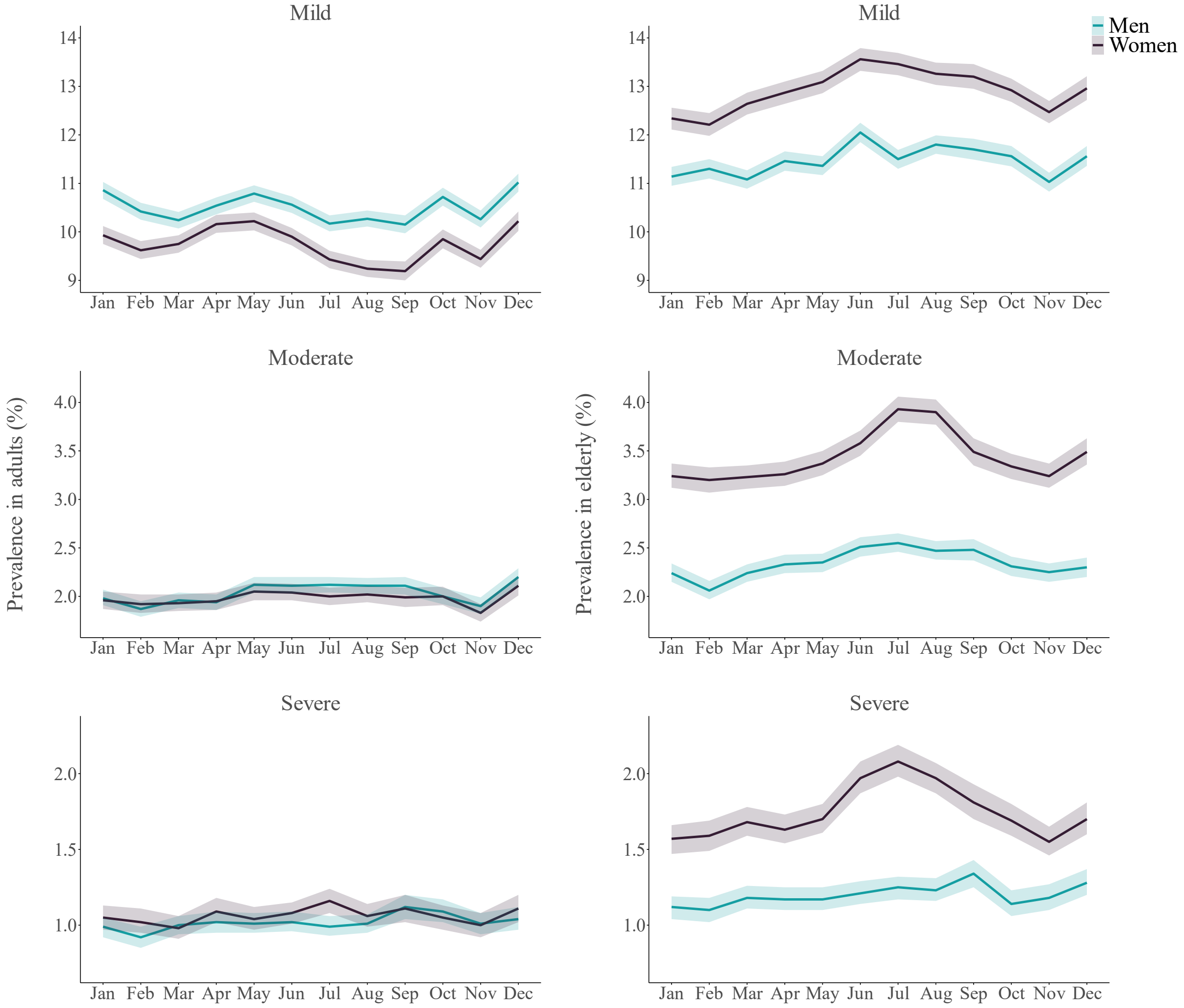

© 2024 Prpic M et al. *JAMA Network Open.*

eFigure 3. Cumulative Risk Ratios (RR) of Hyponatremia due to Increasing Heat Index for Elderly (A), and Adults (B)

A

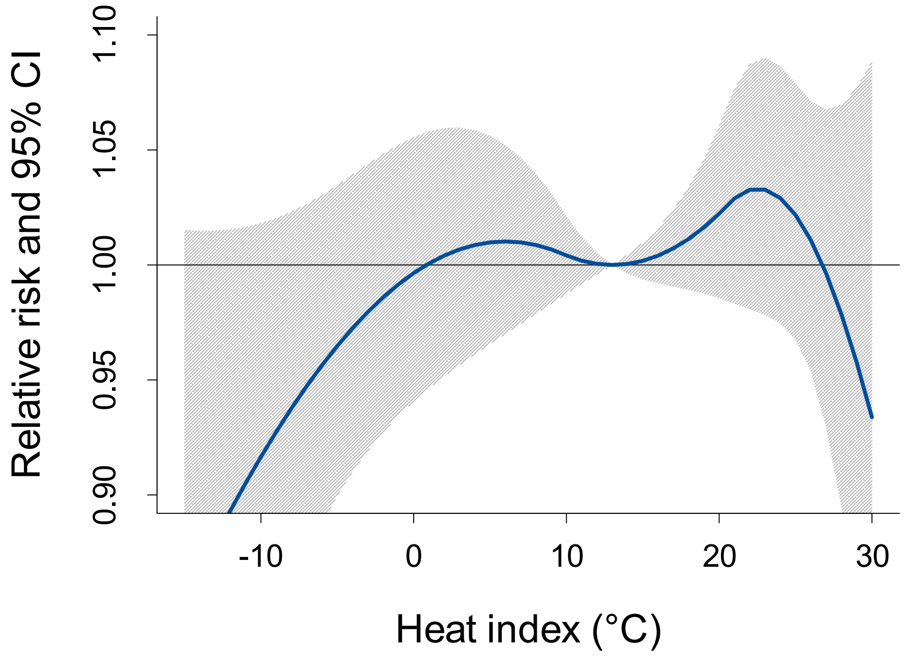

B

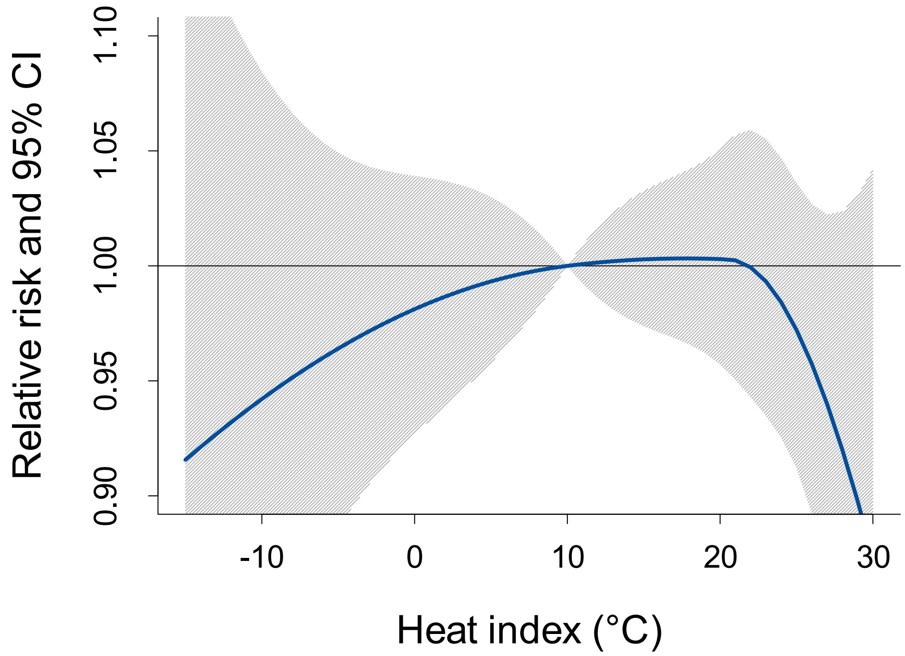

© 2024 Prpic M et al. *JAMA Network Open.*

eFigure 4. Distribution of the Risk Across Lag Days 0 to 5 in Elderly (A), and Adults (B)

A

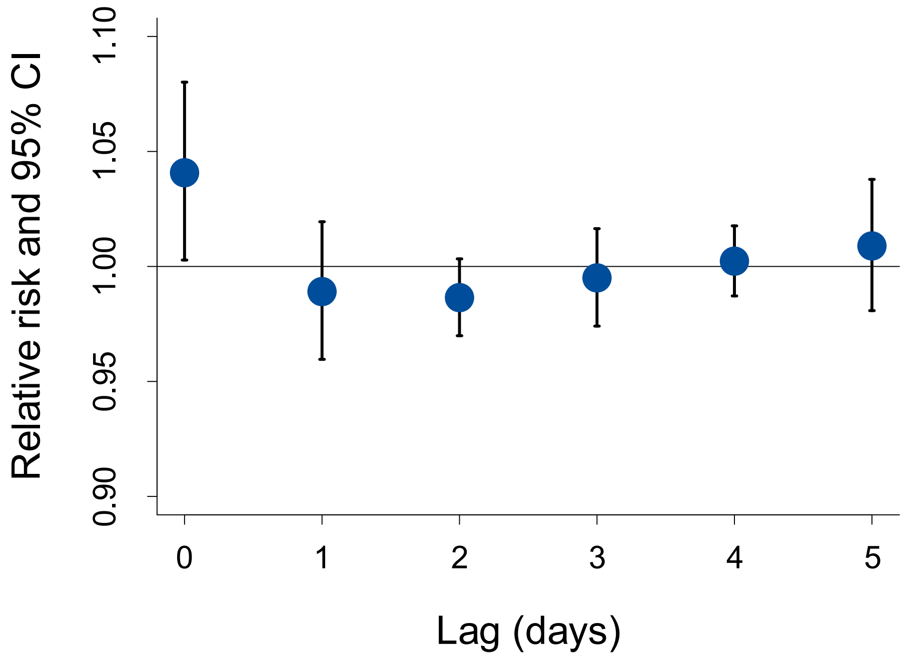

B

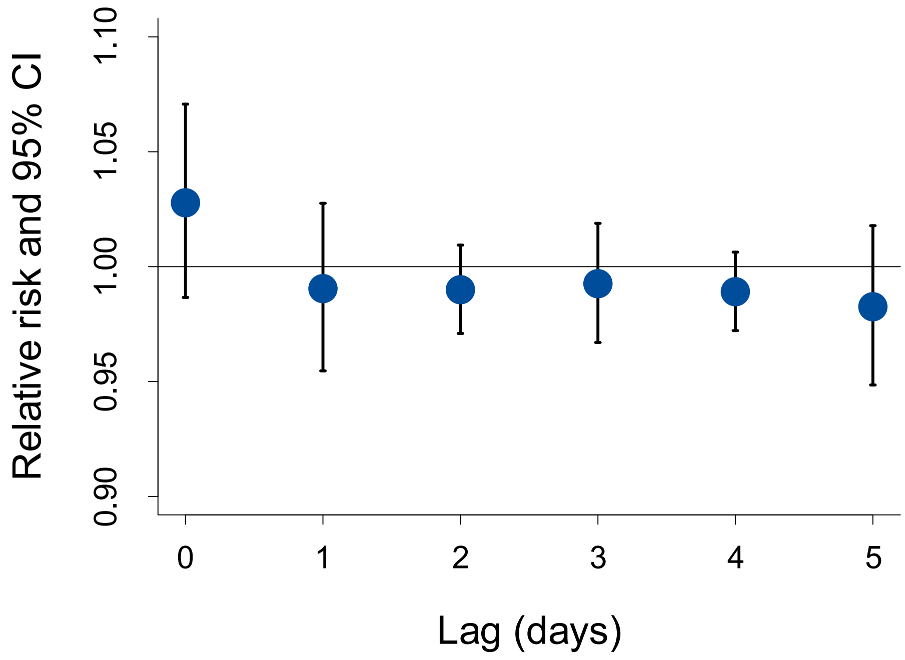

© 2024 Prpic M et al. *JAMA Network Open.*

eTable 1. Monthly Summary of Meteorological Variables and Prevalence Rates

	Jan	Feb	Mar	Apr	May	Jun	Jul	Aug	Sep	Oct	Nov	Dec
**Average temperature, °C**												
Mean (SD)	1·4 (4·7)	2·4 (4·4)	5·5 (4·05)	10·2 (4·1)	14·8 (3·8)	18·7 (3·7)	20·3 (3·4)	19·9 (3·2)	15·5 (3·1)	10·7 (3·4)	6·0 (3·6)	2·6 (4·2)
**Average relative humidity, %**												
Mean (SD)	85·0 (7·6)	79·4 (9·6)	73·0 (12·5)	64·8 (13·0)	64·3 (13·0)	63·1 (12·3)	64·8 (12·8)	67·1 (11·7)	73·4 (10·6)	84·5 (8·5)	86·4 (6·8)	86·3 (7·4)
**Average heat index, °C**												
Mean (SD)	1·1 (4·4)	2·0 (4·0)	4·5 (3·6)	9·1 (4·2)	14·0 (4·0)	18·2 (4·0)	20·0 (3·6)	19·7 (3·4)	15·0 (3·3)	10·0 (3·6)	5·4 (3·3)	2·3 (3·9)
**Overall prevalence, (%)**												
Mild	11.00	10.80	10.80	11.14	11.23	11.35	10.96	10.99	10.89	11.13	10.67	11.34
Moderate	2.17	2.09	2.17	2.18	2.29	2.39	2.45	2.44	2.31	2.22	2.12	2.32
Severe	0.86	0.82	0.88	0.88	0.91	1.01	1.05	1.03	0.95	0.88	0.85	0.91
**Prevalence in adults, (%)**												
Mild	10.44	10.05	10.01	10.36	10.51	10.25	9.81	9.79	9.70	10.31	9.87	10.66
Moderate	1.85	1.79	1.85	1.84	1.97	1.99	1.95	1.97	1.94	1.90	1.76	2.03
Severe	0.80	0.74	0.79	0.80	0.81	0.85	0.85	0.86	0.86	0.83	0.77	0.84
**Prevalence in elderly, (%)**												
Mild	11.67	11.70	11.76	12.08	12.12	12.71	12.35	12.44	12.36	12.14	11.64	12.17
Moderate	2.58	2.47	2.59	2.63	2.72	2.90	3.08	3.03	2.81	2.68	2.61	2.71
Severe	1.09	1.08	1.17	1.14	1.15	1.31	1.41	1.36	1.27	1.12	1.11	1.18

© 2024 Prpic M et al. *JAMA Network Open.*

**Prevalence in adult men, (%)**												
Mild	10.86	10.42	10.24	10.54	10.79	10.56	10.17	10.27	10.15	10.72	10.26	11.02
Moderate	1.98	1.87	1.96	1.94	2.12	2.11	2.12	2.11	2.11	2.00	1.90	2.20
Severe	0.99	0.92	1.00	1.02	1.01	1.02	0.99	1.01	1.12	1.09	1.01	1.04
**Prevalence in adult women, (%)**												
Mild	9.93	9.62	9.75	10.16	10.22	9.90	9.43	9.24	9.19	9.85	9.44	10.22
Moderate	1.96	1.92	1.93	1.95	2.05	2.04	2.00	2.02	1.99	2.00	1.83	2.11
Severe	1.05	1.02	0.98	1.09	1.04	1.08	1.16	1.06	1.11	1.05	1.00	1.11
**Prevalence in elderly men, (%)**												
Mild	11.14	11.30	11.08	11.46	11.36	12.05	11.50	11.80	11.70	11.56	11.03	11.56
Moderate	2.24	2.06	2.24	2.33	2.35	2.51	2.55	2.47	2.48	2.31	2.25	2.30
Severe	1.12	1.10	1.18	1.17	1.17	1.21	1.25	1.23	1.34	1.14	1.18	1.28
**Prevalence in elderly women, (%)**												
Mild	12.34	12.21	12.64	12.87	13.09	13.56	13.46	13.26	13.20	12.92	12.47	12.96
Moderate	3.24	3.20	3.23	3.26	3.37	3.58	3.93	3.90	3.49	3.34	3.24	3.49
Severe	1.57	1.59	1.68	1.63	1.70	1.97	2.08	1.97	1.81	1.69	1.55	1.70

© 2024 Prpic M et al. *JAMA Network Open.*

eTable 2. Sensitivity Analyses of the Relative Risk and 95% Confidence Interval in Elderly, Compared to the Baseline HI at Which Hyponatremia Occurred Least Frequently and Cumulated Over a 5-Day Lag Period (With the Exception of Cases M5 and M6, Where We Adjusted the Number of Lag Days)

Model	Model configuration	Baseline HI	HI with highest RR	RR
M0	Main model	13 °C	23 °C	1.03 [0.98, 1.09]
M1	Excluding years 2020 and 2021 as periods affected with COVID-19 pandemics	13.7 °C	23 °C	1.04 [0.98, 1.10]
M2	Without controlling for comorbidity cofounders	10.5 °C	22 °C	1.04 [0.99, 1.11]
M3	Natural cubic spline with 9 df per year for trend/seasonality control	14.2 °C	22 °C	1.02 [0.97, 1.07]
M4	Cross-basis with natural cubic with 3 internal knots placed at the 10th, 75th and 90th percentiles	12.1°C	22°C	1.03 [0.98, 1.08]
M5	3-day lag period	12.9°C	23°C	1.02 [0.98, 1.07]
M6	7-day lag period	14.1°C	23°C	1.03 [0.97, 1.09]
M7	Hyponatremia defined on person-encountered level instead of on person-day level	11.7 °C	23 °C	1.06 [1.01, 1.11]

© 2024 Prpic M et al. *JAMA Network Open.*
