# Supplementary material for: Urban Heat and Burden of Hyponatremia
Source: JAMA Netw Open. 2024 Dec 16;7(12):e2450280. doi: 10.1001/jamanetworkopen.2024.50280 (PMC11650395; doi:10.1001/jamanetworkopen.2024.50280)
Supplement: Supplement 2. — Data Sharing Statement [file jamanetwopen-e2450280-s002.pdf]

Data Sharing Statement

Prpic. Urban Heat and Burden of Hyponatremia. *JAMA Netw Open*. Published December 16, 2024. doi:10.1001/jamanetworkopen.2024.50280

Data
Data available: No

Additional Information
Explanation for why data not available: Data supporting this study contain sensitive personal data and cannot be made publicly available due to ethical and legal restrictions. Requests for aggregated data may be considered on a case-by-case basis by contacting the corresponding author kai.kappert@charite.de.
